# Trials and tribulations of diagnosing and managing psychosis secondary to non-convulsive epilepsy

**DOI:** 10.1192/bjo.2021.334

**Published:** 2021-06-18

**Authors:** Mahmoud Awara, Joshua Smalley, Matt Havenga, Manal Elnenaei

**Affiliations:** 1 Dalhousie; Nova Scotia Health Authority; Royal College of Psychiatrists; 2 Maastricht University; Child and Adolescent Psychiatrist at Peterborough Regional Health Centre; 3 Dalhousie University, Nova Scotia Health Authority

## Abstract

**Objective:**

To highlight the importance of reviewing diagnosis and management of refractory psychosis and to share that with the scientific community; and to also shed some light on the dilemma and challenges that professionals may face to diagnose and treat organic psychosis. In addition, to look at the possible similarity/dissimilarity in psychopathology between organic and primary psychosis and differences in opinions through presenting the history and course of illness of this patient.

**Case report:**

We present the case of a 51-year-old female who had a 28-year history of treatment-resistant schizophrenia. She did not report or display any seizure activity, and an extensive investigation was unremarkable. The unusual nature of her psychopathology, which was predominantly visual hallucinations and somatic delusions, and the difficult to treat nature of her symptoms, prompted investigation with Electroencephalograph which demonstrated bilateral temporal lobe epileptic activity.

**Discussion:**

Treatment with divalproex sodium and discontinuation of antipsychotic medication achieved an excellent response, where her visual hallucinations and somatic delusions were both remarkably ameliorated.

**Conclusion:**

The differentiation between organic/secondary and functional/primary psychosis is an area of contention between psychiatrists and neurologists and also within each of these specialties.

The myriad of psychopathology and associated treatment resistant psychotic symptoms that patients with non-convulsive epilepsy may experience should result in building a long desired bridge between neurology and psychiatry to collaborate in managing such cases.

